# A Tale of Two: 2-Year Outcomes of 2 Patients Undergoing Compassionate Use INTREPID and TricValve System Placement: Case Series

**DOI:** 10.1016/j.jscai.2025.103705

**Published:** 2025-05-01

**Authors:** Hernan L. Vera-Sarmiento, Tsuyoshi Kaneko, Alan Zajarias

**Affiliations:** a Internal Medicine Residency Program, Washington University School of Medicine, St Louis, Missouri; b Division of Cardiothoracic Surgery, Washington University School of Medicine, St. Louis, Missouri; c Cardiovascular Division, Washington University School of Medicine, St. Louis, Missouri

**Keywords:** case report, transcatheter tricuspid valve repair, tricuspid regurgitation, TricValve

## Abstract

Tricuspid regurgitation contributes to worsening heart failure symptoms, quality of life, and mortality. Medical therapy provides limited benefit, and surgical intervention in high-risk patients is associated with significant postoperative complications. Transcatheter tricuspid valve interventions are improving, but until recently, patient candidacy has been limited by the stringent inclusion and exclusion criteria of clinical trials. We present 2 cases of patients with severe tricuspid regurgitation treated in a compassionate use setting with transcatheter therapies. Patient 1 underwent TricValve (Products & Features) placement for mitigation of right-sided heart failure with at least 24 months free of hospitalization. Patient 2 underwent compassionate INTREPID valve (Medtronic) placement with at least 24 months free of heart failure-related hospitalization.

## Introduction

Tricuspid regurgitation (TR) affects about 0.55% of the US population and significantly worsens quality of life.^1^^,^^2^ It is associated with poor clinical outcomes and mortality, independent of pulmonary pressures and heart failure (HF).^3^ Although yet to show mortality benefits, novel transcatheter therapies have shown improvement in New York Heart Association (NYHA) class and quality of life, as well as lower hospitalization rates.^4^^,^^5^ Eligibility criteria for inclusion in clinical trials evaluating these therapies can be restrictive, and many patients failing medical therapy are left with limited options. We present 2 cases of compassionate use transcatheter tricuspid valve therapies that provided benefit to otherwise trial ineligible patients.

## Clinical cases

### Case 1

An 89-year-old woman with chronic kidney disease stage 4, hypertension, severe mitral regurgitation (MR), HF with preserved ejection fraction, massive TR, and new paroxysmal atrial fibrillation presented with 2 months of worsening fatigue, lower extremity edema, dyspnea on exertion, and NYHA class III to IV symptoms despite adherence to medical management. Right heart catheterization demonstrated ventricularized right atrial pressures, preserved right ventricular (RV) function, and low cardiac index (Table 1). Transesophageal echocardiogram revealed normal left ventricular function, severe MR due to calcific mitral valve disease not eligible for transcatheter edge to edge repair, dilated RV with decreased function, and massive TR due to annular dilation (Supplemental Videos 1 and 2).

**Table 1:** Transthoracic echocardiogram and right heart catheterization results for cases 1 and 2.

	Case 1	Case 2
TTE		
LVEF	81%	70% and reduced VTI SV
RV dilation	Marked RV dilation	Massive RV dilation and pHTN
TR/MR	Massive TR and severe MR with calcified annulus	Torrential TR with new flail anterior leaflet
RHC		
Mean RAP, mm Hg	22	17
RVP, mm Hg	36/15	53/15
PAP, mm Hg	45/19/28	62/30/43
Cardiac index, L/min/m^2^	1.85	2

LVEF, left ventricular ejection fraction; MR, mitral regurgitation; PAP, pulmonary artery pressure; pHTN, pulmonary hypertension; RAP, right atrial pressure; RHC, right heart catheterization; RV, right ventricle; RVP, right ventricular pressure; TR, tricuspid regurgitation; TTE, transthoracic echocardiogram; VTI SV, velocity time integral stroke volume.

In a multidisciplinary meeting, the patient was considered at extreme-risk for surgery due to her comorbidities. She was not considered mitral transcatheter edge to edge repair eligible due to small valve area and leaflet calcification. She was not a candidate for transcatheter mitral valve replacement due to risk of left ventricular outflow tract obstruction, which, combined with massive TR, led to her exclusion from ongoing clinical trials. Attempts to obtain compassionate use approval for mitral therapies were unsuccessful due to the perceived short-term mortality risk in this patient population with severe bivalvular disease. The patient was deemed ineligible for available TR clinical trials because of severe MR. Given the limited treatment options, the patient continued medical therapy for 6 months, during which she experienced worsening lower extremity edema, abdominal swelling, and fatigue.

**Figure 1:**
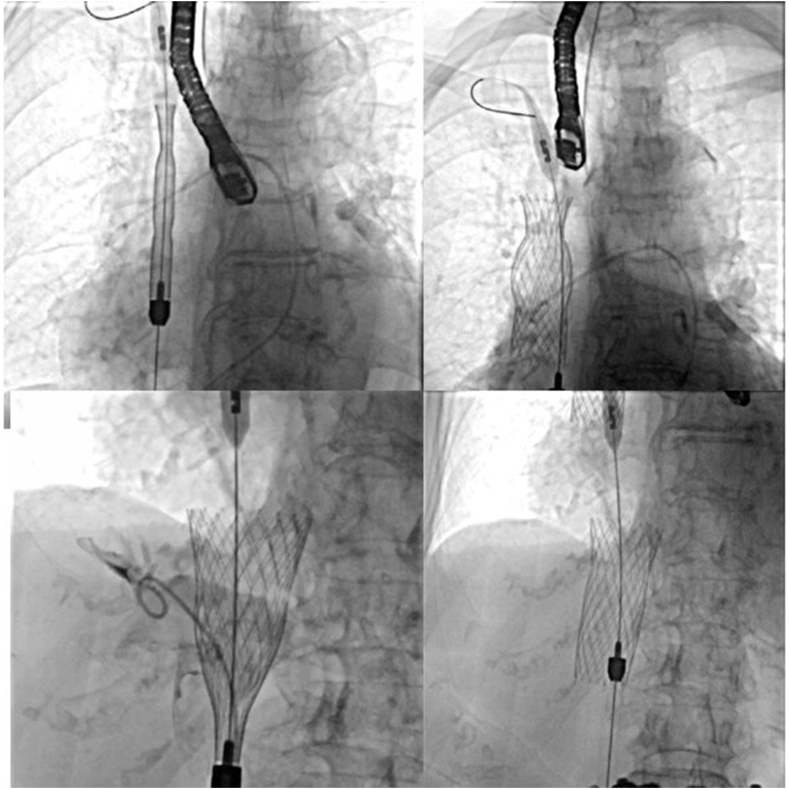
**TricValve placement with a 25- and 41-mm frame**.

Due to her worsened systemic congestion symptoms, compassionate use TricValve (Products & Features) was requested. The patient underwent placement in July 2022 without complications (Figure 1). Postprocedural transthoracic echocardiogram (TTE) confirmed normal functioning caval prostheses. At 6-, 12-, and 24-month follow-up, the patient remained NYHA functional class II and remained hospitalization free.

### Case 2

**Figure 2:**
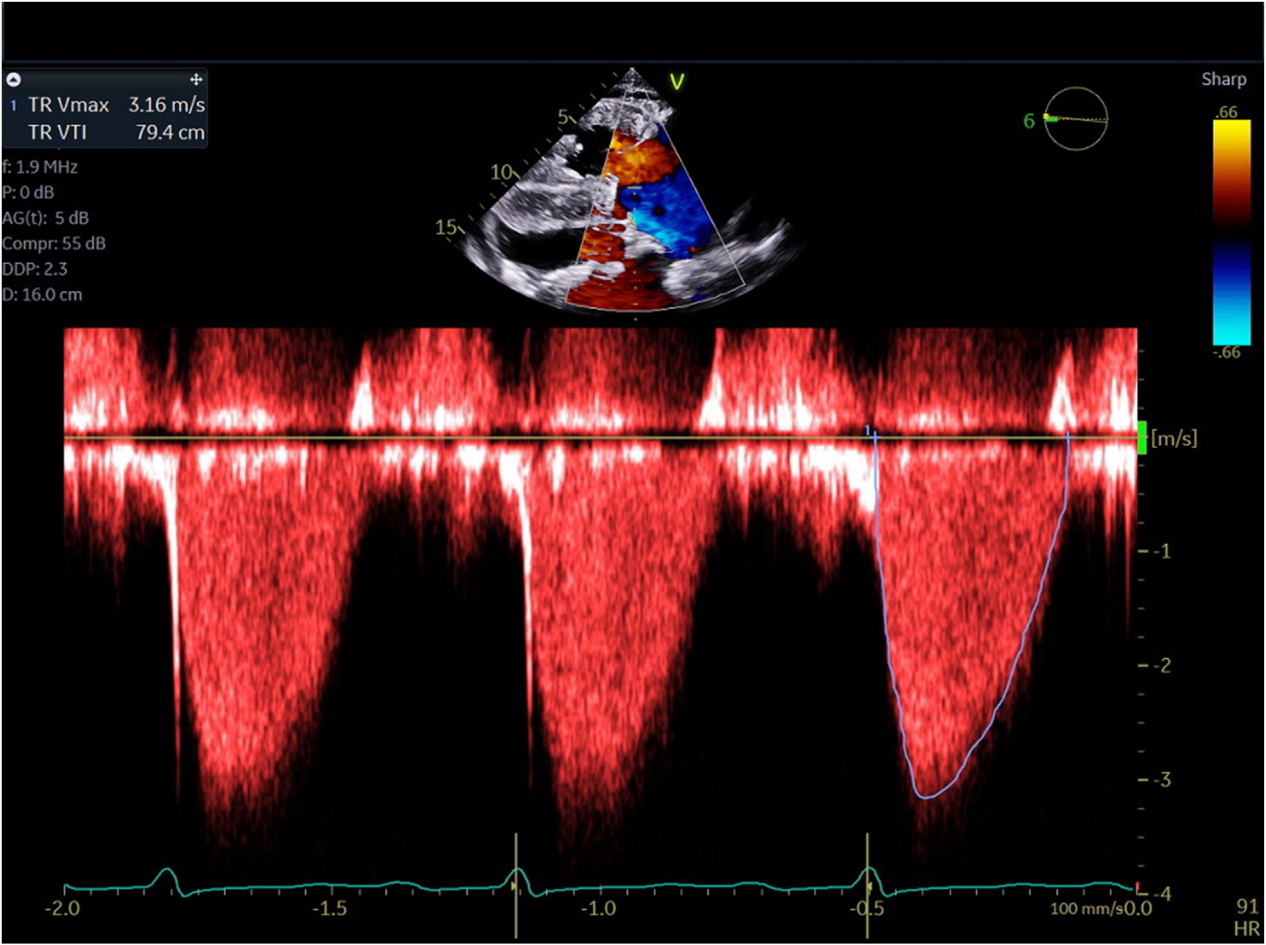
**Patient 2 preintervention transthoracic echocardiogram demonstrating torrential tricuspid regurgitation and flail leaflet**.

A 44-year-old African American woman with idiopathic pulmonary arterial hypertension on intravenous therapy, right-sided HF, and atrial fibrillation presented with NYHA class III to IV symptoms. She complained of abdominal distention and weight gain 3 weeks after tunneled infusion catheter exchange. Her TTE revealed RV dilation, severe pulmonary hypertension (pHTN), and new torrential TR secondary to a flail anterior leaflet (Table 1; Figure 2).

**Figure 3:**
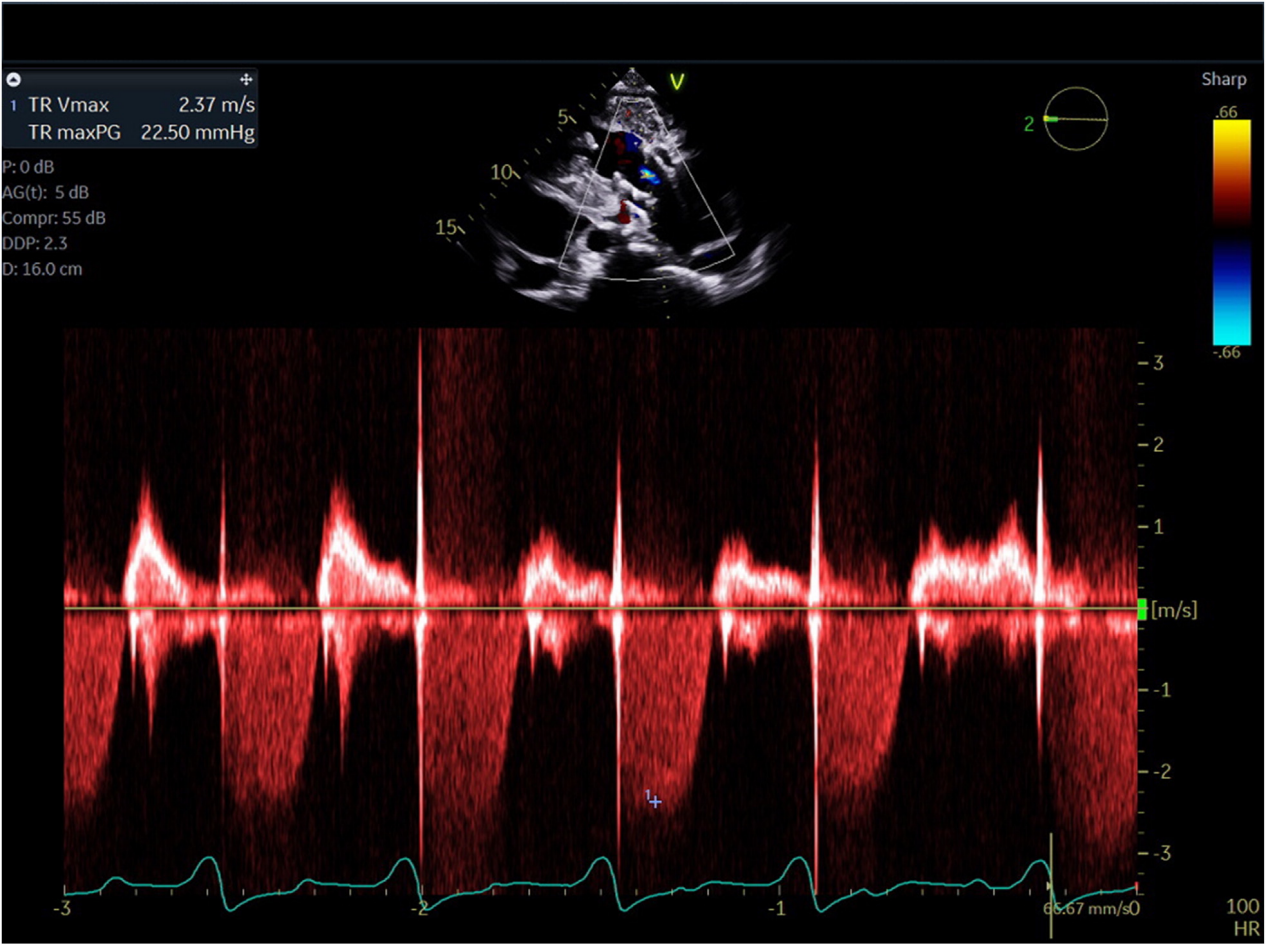
**Post INTREPID valve placement transthoracic echocardiogram with tricuspid regurgitation resolution**.

In a multidisciplinary meeting, the patient was deemed ineligible for surgical valve replacement or transcatheter therapy trials due to tricuspid anatomy and severe pHTN. Despite medical therapy adjustment, the patient had multiple hospitalizations for worsening symptoms in a short time span, which led to risk/benefit discussion about compassionate use devices. Due to the loss of integrity of the tricuspid valve, a valve prosthesis that would anchor independently of the valve leaflets was pursued. The patient underwent compassionate use transcatheter tricuspid valve replacement with the 48 mm INTREPID system (Medtronic) in July 2021 (Supplemental Video 3). Repeat TTE demonstrated adequate valve placement and TR resolution (Figure 3).

At 6-, 12-, and 24-month follow-up, patient remained NYHA functional class II, remained HF hospitalization free, and has returned to work.

## Discussion

In high- or extreme-risk patients with bivalvular disease, it is important to identify the predominant valve lesion and focus therapy on it. Patients with concomitant severe MR and TR are excluded from clinical trials to maintain a homogeneous patient sample and assess the efficacy of the device being evaluated.^4^^,^^5^ In case 1, the patient had severe MR with preserved left ventricular function, and the TR appeared to be the predominant lesion, as her symptoms were primarily related to systemic congestion. The TricValve prosthetic relieved the symptoms without interfering with RV function and provided the expected favorable results for more than 2 years. The TRICAV-II and TRICAV-II trials (NCT06137807) are designed to evaluate patients with severe TR utilizing the TricValve system in patients ineligible for current US Food and Drug Administration-approved therapies and are actively enrolling.^6^

Severe pHTN has been considered an exclusion for the treatment of severe TR.^7^ Understanding the mechanism of TR is critical to determining patient eligibility for therapy. The most common mechanism of TR in patients with pulmonary arterial hypertension is annular enlargement and leaflet tethering. In case 2, the patient experienced a degenerative process (ruptured chord with a flail leaflet) that precipitated the TR and clinical deterioration. Since the mechanism was not functional and the right ventricle did not show dysfunction, addressing the valve pathology was beneficial and led to a positive outcome.

## Conclusion

The field of transcatheter tricuspid valve interventions is still in its infancy. Applying the correct diagnostic tools allow us to better understand patient eligibility for transcatheter interventions. As we learn the benefits and limitations of repair or replacement, orthotopic, or heterotopic procedures, we can tailor their use to address the individual needs of our patients.
